# Clinical application of artificial intelligence algorithms in detecting clival remodeling in the setting of pituitary neuroendocrine tumors/pituitary adenomas

**DOI:** 10.3389/fneur.2026.1697911

**Published:** 2026-04-10

**Authors:** Diego D. Luy, Mousa Javidialsaadi, Andre Payman, Faraz Behzadi, Joseph F. Zywiciel, Andrew Pickles, Joseph Frazzetta, Isaac Ng, Shiau-Sing Cicierska, Vikram C. Prabhu, Chirag Patel, Anand V. Germanwala

**Affiliations:** 1 Department of Neurological Surgery, Loyola University Medical Center, Maywood, IL, United States; 2 Stritch School of Medicine, Loyola University Chicago, Chicago, IL, United States

**Keywords:** artificial intelligence, clivus, machine learning, pituitary neuroendocrine tumors, skull base

## Abstract

**Introduction:**

Pituitary neoplasms may expand the sella and invade into adjacent structures, including the sphenoid sinus and/or the clivus. Previously, sellar remodeling assisted with detecting these tumors prior to the creation of computed tomography and magnetic resonance imaging. This project aims to quantify efficacy for discerning clival osseous changes in patients with pituitary neuroendocrine tumors (PitNETs) when compared to controls using artificial intelligence and machine learning models.

**Methods:**

Electronic health records were reviewed. Still images of standard bone window CT heads were captured and compared using supervised machine learning/convolutional neural network (CNN) models trained on three singular axis (axial, coronal, or sagittal) CT sequences of a manually segmented clivus bone for each patient (102 images from 34 functioning PitNETs, 240 images from 80 nonfunctioning PitNET, and 387 images from 129 normal patients).

**Results:**

Overall, accuracies were favorable for axial sequences: Model 1 (axial PitNET vs. normal, accuracy 81%) and Model 4 (axial non-functioning PitNET vs. functioning PitNET, accuracy 95%), and Model 7 (axial non-functioning PitNET vs. functioning PitNET vs. normal, accuracy 83%). This performance difference may be due to added benefit of bilateral and anterior–posterior image features on axial views. Although this bilaterality of information is also available in coronal views, models consistently performed poorly compared to sagittal and axial sequences.

**Discussion/conclusion:**

To date, no reports have detailed use of a CNN to identify subtle osseous changes and potentially detect PitNETs based on CT bone windows alone. Our models produced average accuracies up to 81% in correct identification of PitNET vs. control and 95% correct identification of functioning vs. nonfunctioning PitNET. These findings serve as a proof-of-concept that CNNs, may be trained to provide acceptable levels of accuracy with CT imaging, a modality more readily available than MRI.

## Introduction

Pituitary neoplasms frequently expand the sella turcica and may invade into adjacent structures, including the sphenoid sinus anteroinferiorly and/or the clivus posteroinferiorly (1–5). Interestingly, identification of sellar remodeling has proven effective as a diagnostic method to detect these tumors prior to the advent of computed tomography and magnetic resonance imaging (1). To date, no reports have detailed the potential role for identifying clival osseous changes with artificial intelligence (AI) and/or machine learning (ML) algorithms, although, these algorithms have previously been used to aid surgeons with predicting cerebrospinal fluid leaks prior to skull base surgery (6). This project aims to quantify efficacy for discerning clival osseous changes in patients with pituitary neuroendocrine tumors (PitNETs) when compared to controls with no sellar/skull base pathology. Additionally, these findings may be expanded in the future to correlate osseous changes to tumor characteristics (size, histopathological, location) and postoperative patient outcomes (visual impairment, hormone derangements, length of stay).

## Materials and methods

Electronic health records for adult patients diagnosed with functioning and nonfunctioning pituitary neuroendocrine tumors treated at a single center from 2008–2025 were reviewed. Control patients without skull-base and/or intracranial neoplasms were included (Table 1). Exclusion criteria for the PitNETs included sellar surgery prior to computed tomography (CT) head imaging. Still images of standard bone windows with equal window width: 2,500 and level 480 Hounsfield units for non-contrast and contrasted CT heads were exported as portable network graphics (PNG) images while prioritizing the most demonstrative and least artifactual representation of the clivus; images were then manually segmented by one designated data collector to isolate the clivus bone in axial, coronal, and sagittal views for controls and PitNETs patients. If images were limited by artifact or patient mispositioning in the CT scanner, they were excluded from the study. Data was deidentified prior to storage and analysis. Ethical oversight was obtained through our institutional research board (Reference Number 219408). The institutional research board determined the study met criteria for exempt status as no identifiable information was collected or analyzed and individual informed consent was not required for completion of this retrospective human imaging observational study.

**Table 1:** Clinical characteristics summary.

Study characteristics	Normal	Functioning PitNET	Non-functioning PitNET
Patients (*n*)	129	34	80
Age (years)			
Median	64	43.5	58.5
Range	18–99	19–81	29–87
Sex (M/F)	58/71	13/21	53/27
Extent of resection			
Gross total resection	—	27	62
Subtotal resection	—	7	18
Pituitary apoplexy	—	2	21
Adherent tumor	—	9	15
Firm tumor	—	3	6
Intraoperative CSF leak	—	9	26
Postoperative need for HRT	—	21	40
Postoperative DI	—	5	13
Recurrence	—	1	9

Functioning PitNET includes tumors secreting demonstrated variable expression of prolactin, growth hormone, adrenocorticotropic hormone, thyroid-stimulating hormone, follicle-stimulating hormone, luteinizing hormone, and synaptophysin.

PitNET, pituitary neuroendocrine tumor; CSF, cerebrospinal fluid; HRT, hormone replacement therapy; DI, diabetes insipidus.

CT images were obtained along the axial, coronal, and sagittal planes of the clivus for functioning (*n* = 34) and nonfunctioning (*n* = 80) PitNET patients and non-tumor control (*n* = 129) patients. Bone window width 2,500 and level 480 Hounsfield units were used for these images. Functioning PitNET demonstrated variable expression of prolactin, growth hormone, adrenocorticotropic hormone, thyroid-stimulating hormone, follicle-stimulating hormone, luteinizing hormone, and synaptophysin. Images were then manually segmented to isolate the clivus bone for each patient. One image per patient per view (axial/coronal/sagittal) was collected. A total of 102 images from 34 functioning PitNETs, 240 images from 80 nonfunctioning PitNET, and 387 images from 129 normal patients were used to train the nine models created.

Python 3.7 with open-source libraries (numpy, torch, torchvision, sklearn, matplotlib, seaborn, pytorch_grad_cam) were imported prior to dividing datasets into training (70%), validation (15%), and test (15%) sets. Two complementary deep learning model architectures were used; ResNet-18 convolutional neural network (CNN) allowed for residual skip connections to complete efficient deep feature extraction while minimizing vanishing effects while Vision Transformer (ViT) allowed for division of images into patches to establish global spatial relationships not readily captured by CNNs. Models created with these architectures were trained using a standard method for refining model parameters (stochastic gradient descent with momentum) to minimize classification error (cross-entropy loss) for these categorical prediction tasks. Smoothed class weights were also applied and incorporated into the cross-entropy loss function to limit impact of class imbalance. Training over several cycles (epochs) were completed on the primary dataset with periodic evaluation of performance on a separated validation set as listed above. To avoid overtraining/bias towards the training set (overfitting), training was halted once the model’s performance on classification of the validation set could not be improved, thereby yielding the best-performing version of this model. Overfitting was also limited through data augmentation of the training set where images were randomly rotated 10 degrees, had mild brightness/contrast variations of 10%, and random horizontal flipping. Data augmentation was not applied to the validation images. For ViT models, pretrained ViT-base architecture was used. Given our limited number of patients/images, precautions were taken to avoid overfitting including initiating all ViT models with ImageNet-pretrained weights, reducing learning rate to 3 × 10^−5^, applying weight decay of 1 × 10^−4^, and early stopping similar to those of CNN above (7, 8).

Model performance on unseen test sets were evaluated using accuracy of correct classification (normal vs. PitNET; non-functioning PitNET vs. functioning PitNET; normal vs. non-functioning PitNET vs. functioning PitNET), precision, recall, and *F*_1_-scores. Confusion matrices and ROC curves were created to assist in visualizing true vs. false positives and negatives. gradient-weighted class activation mapping (Grad-CAM) was then applied to assist with interpretability as heat maps demonstrated image features contributing to classification decisions.

## Results

**Figure 1:**
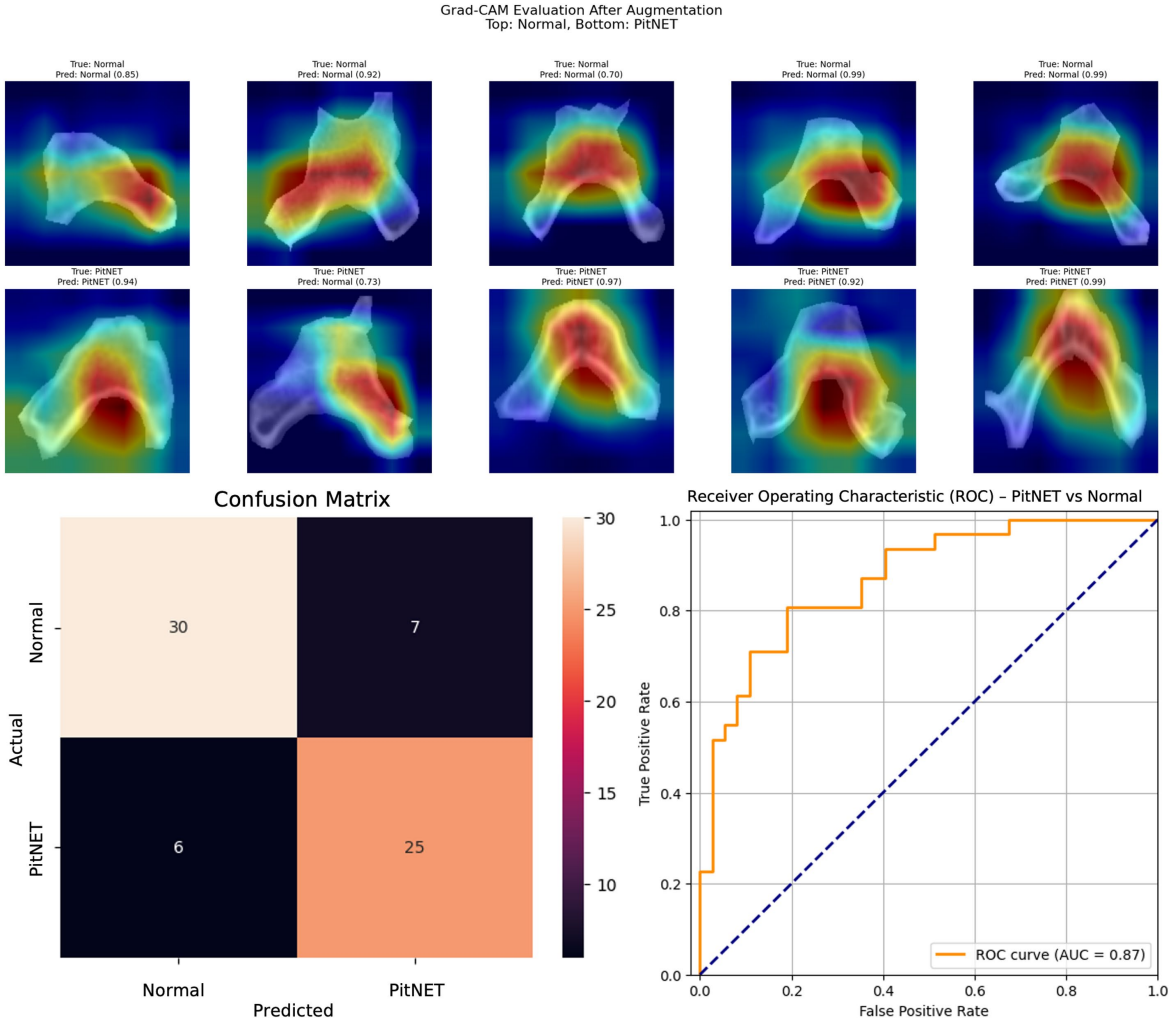
GRAD-CAM visualizations of CNN model emphasis in classification of groups, confusion matric, and ROC curve for Model 1 (axial PitNET vs. normal).

**Figure 2:**
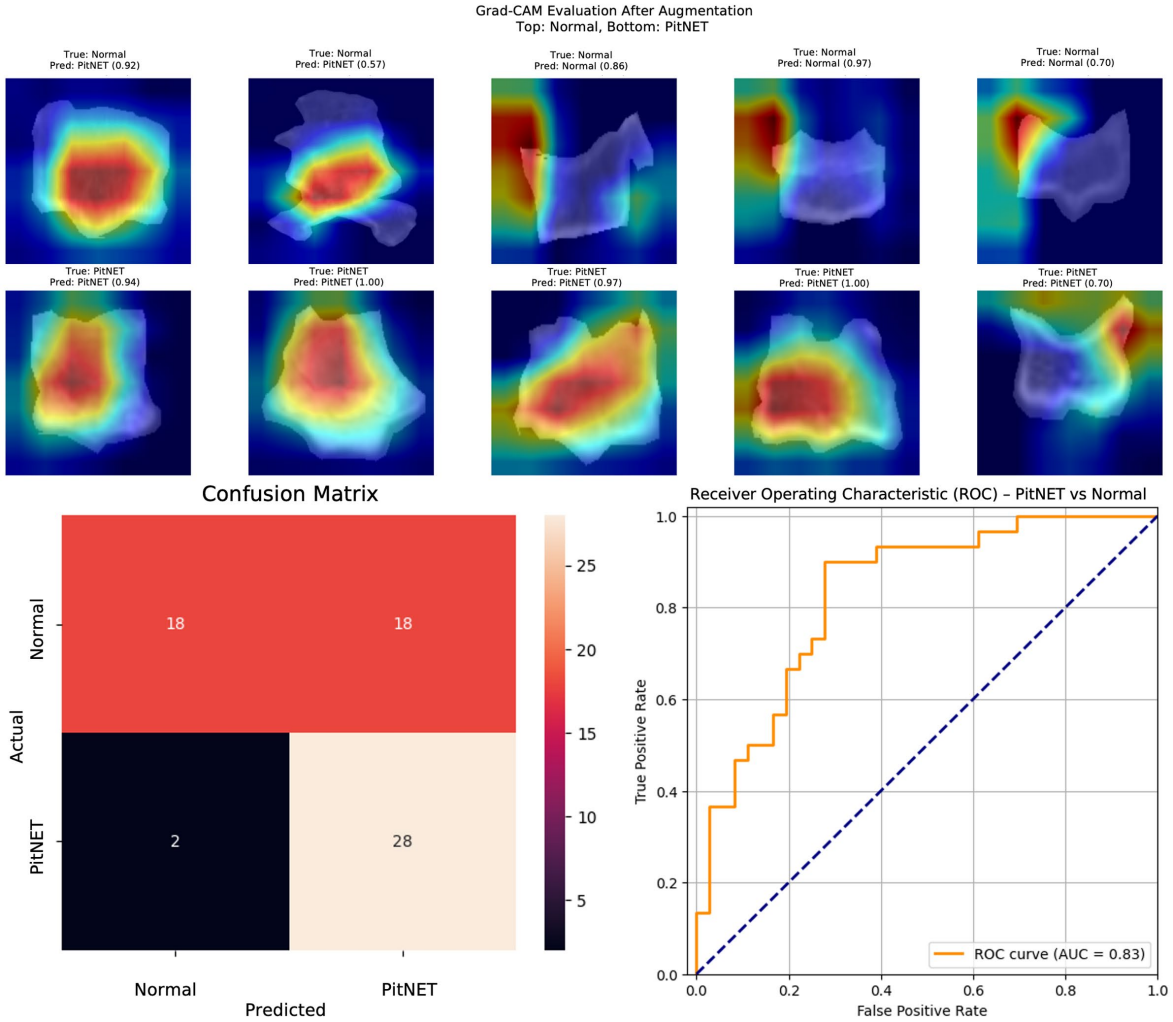
GRAD-CAM visualizations of CNN model emphasis in classification of groups, confusion matric, and ROC curve for Model 2 (coronal PitNET vs. normal).

**Figure 3:**
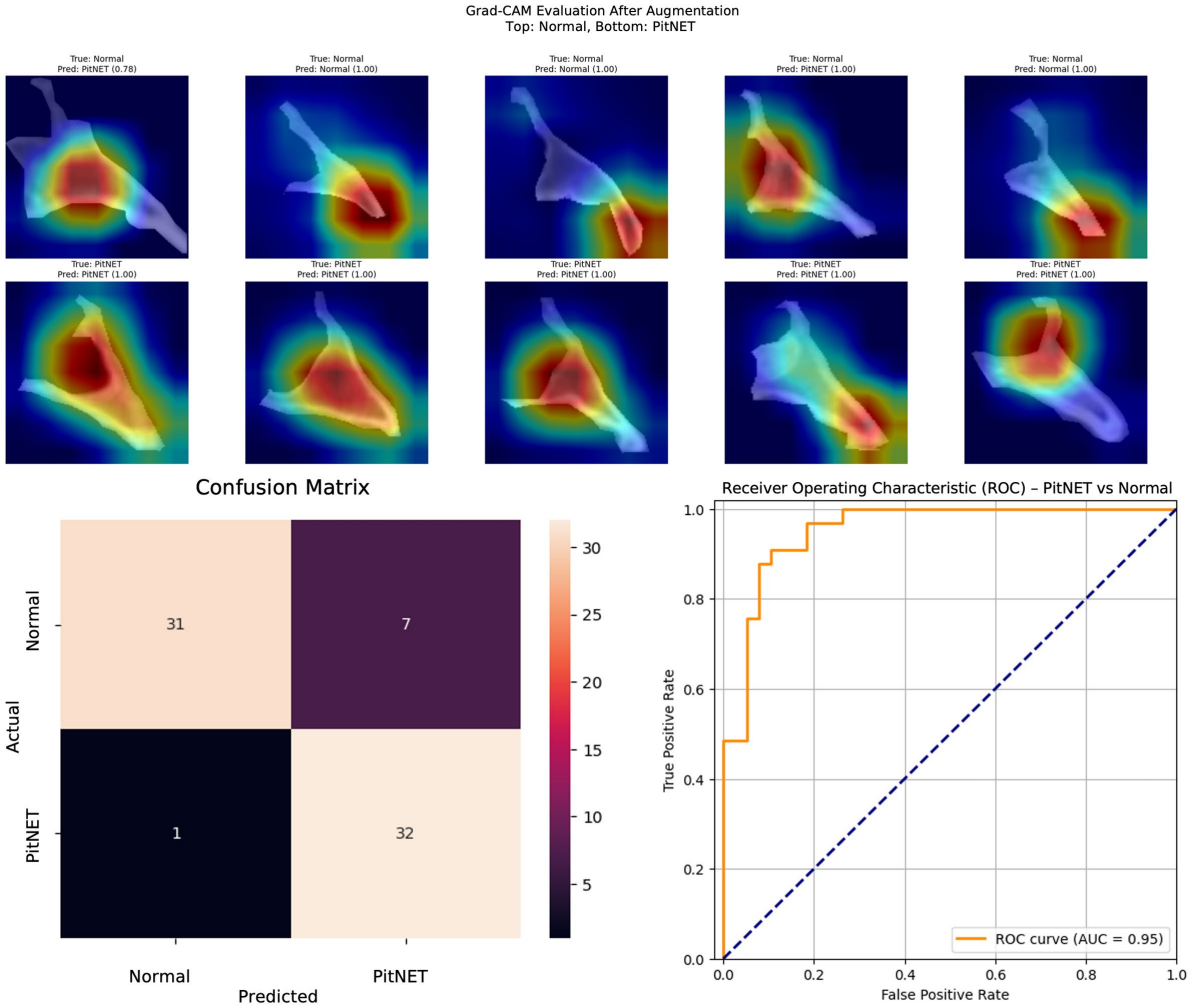
GRAD-CAM visualizations of CNN model emphasis in classification of groups, confusion matric, and ROC curve for Model 3 (sagittal PitNET vs. normal).

Segmented images of bony window CT images of the clivus bone were used to train nine convolutional neural network models (Table 2). Models 1–3 evaluated presence versus absence of PitNET based on still images of the clivus across axial, coronal, or sagittal axes. Model 1 (axial PitNET vs. normal, Figure 1) demonstrated a prediction accuracy of 0.81, area under curve (AUC) 0.87 with *F*_1_-scores of 0.82 (normal) and 0.79 (PitNET). Model 2 (coronal PitNET vs. normal, Figure 2) resulted in an accuracy of 0.70, AUC 0.83, with *F*_1_-scores 0.64 (normal) and 0.74 (PitNET). Model 3 (sagittal PitNET vs. normal, Figure 3) had an accuracy of 0.89, AUC 0.95, *F*_1_-scores of 0.89 (normal) and 0.89 (PitNET).

**Table 2:** Supervised artificial intelligence machine learning model performances by training features.

Model	Features	Average accuracy	*F*_1_-scores	AUC
1	PitNET vs. normal (axial)	0.81 (95% CI 0.71–0.90)	0.82 (95% CI 0.71–0.91; normal)	0.87 (95% CI 0.78–0.95)
			0.79 (95% CI 0.68–0.89; PitNET)	
2	PitNET vs. normal (coronal)	0.70 (95% CI 0.59–0.80)	0.64 (95% CI 0.49–0.78; normal)	0.83 (95% CI 0.73–0.92)
			0.74 (95% CI 0.61–0.84; PitNET)	
3	PitNET vs. normal (sagittal)	0.89 (95% CI 0.82–0.96)	0.89 (95% CI 0.81–0.96; normal)	0.95 (95% CI 0.90–0.99)
			0.89 (95% 0.81–0.96; PitNET)	
4	Non-functioning PitNET vs. functioning PitNET (axial)	0.95 (95% CI 0.87–1.00)	0.96 (95% CI 0.89–1.00; non-functioning PitNET)	0.97 (95% CI 0.91–1.00)
			0.91 (95% CI 0.74–1.00; functioning PitNET)	
5	Non-functioning PitNET vs. functioning PitNET (coronal)	0.82 (95% CI 0.68–0.92)	0.86 (95% CI 0.74–0.95); non-functioning PitNET)	0.91 (95% CI 0.79–0.98)
			0.74 (95% CI 0.50–0.90; functioning PitNET)	
6	Non-functioning PitNET vs. functioning PitNET (sagittal)	0.71 (95% CI 0.56–0.83)	0.76 (95% CI 0.62–0.87; non-functioning PitNET)	0.87 (95% CI 0.74–0.98)
			0.62 (95% CI 0.40–0.79; functioning PitNET)	
7	Non-functioning PitNET vs. functioning PitNET vs. normal (axial)	0.83 (95% CI 0.74–0.91)	0.84 (95% CI 0.72–0.92; non-functioning PitNET)	0.92 (95% CI 0.86–0.97)
			0.62 (95% CI 0.31–0.86; functioning PitNET)	
			0.87 (95% CI 0.80–0.94; normal)	
8	Non-functioning PitNET vs. functioning PitNET vs. normal (coronal)	0.76 (95% CI 0.66–0.84)	0.75 (95% CI 0.61–0.85; non-functioning PitNET)	0.91 (95% CI 0.86–0.96)
			0.54 (95% CI 0.27–0.78; functioning PitNET)	
			0.82 (95% CI 0.73–0.90; normal)	
9	Non-functioning PitNET vs. functioning PitNET vs. normal (sagittal)	0.72 (95% CI 0.62–0.81)	0.69 (95% CI 0.54–0.82; non-functioning PitNET)	0.89 (95% CI 0.84–0.94)
			0.39 (95% CI 0.11–0.67; functioning PitNET)	
			0.80 (95% CI 0.71–0.88; normal)	

PitNET, pituitary neuroendocrine tumor; CNN, convolutional neural network; AI, artificial intelligence; ML, machine learning; ViT, Vision Transformer; CT, computed tomography; Grad-CAM, gradient-weighted class activation mapping; NFPitNET, non-functioning pituitary neuroendocrine tumor; FPitNET, functioning pituitary neuroendocrine tumor; CSF, cerebrospinal fluid; HRT, hormone replacement therapy; DI, diabetes insipidus.

**Figure 4:**
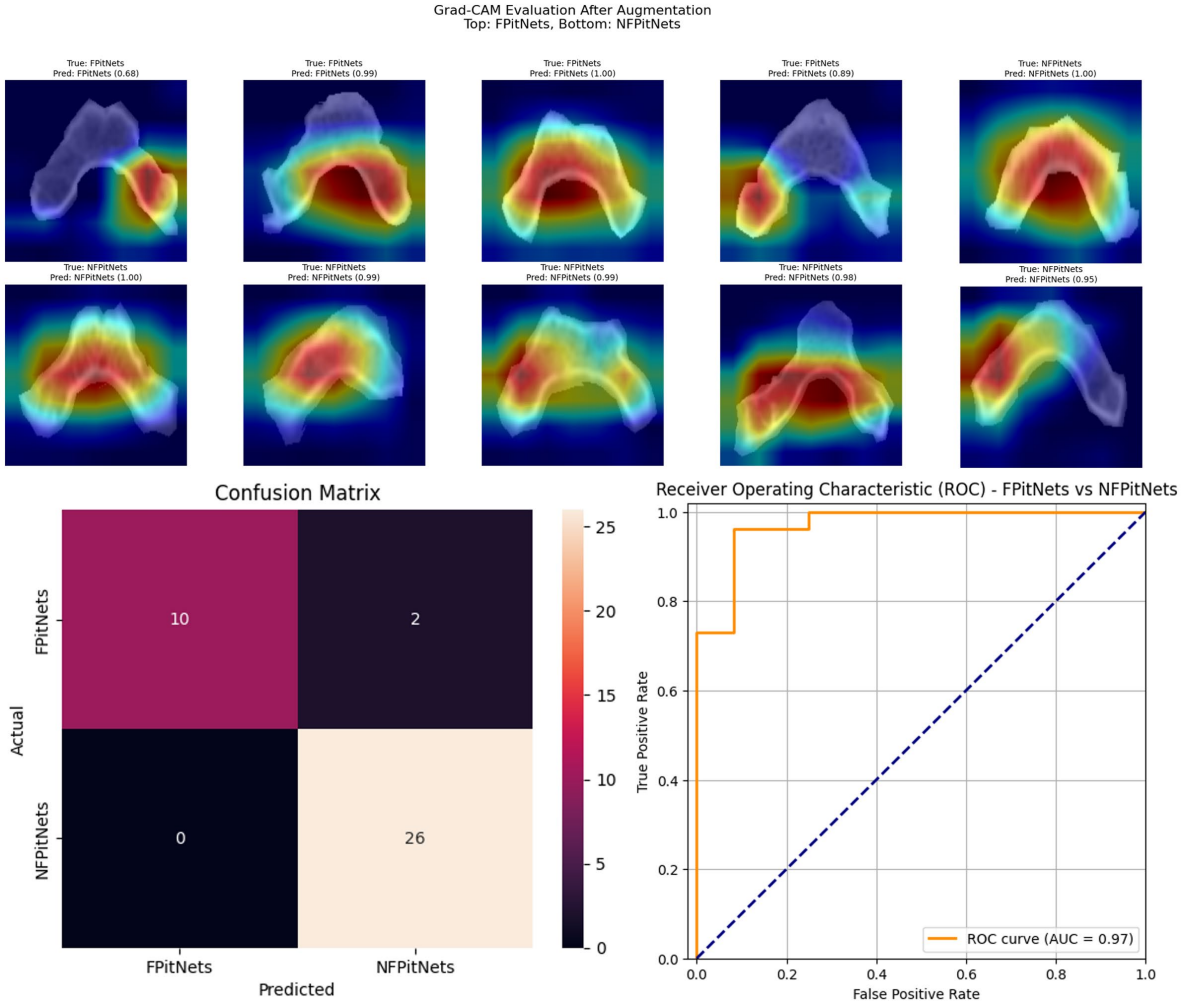
GRAD-CAM visualizations of CNN model emphasis in classification of groups, confusion matric, and ROC curve for Model 4 (axial functional PitNET vs. nonfunctional PitNET).

**Figure 5:**
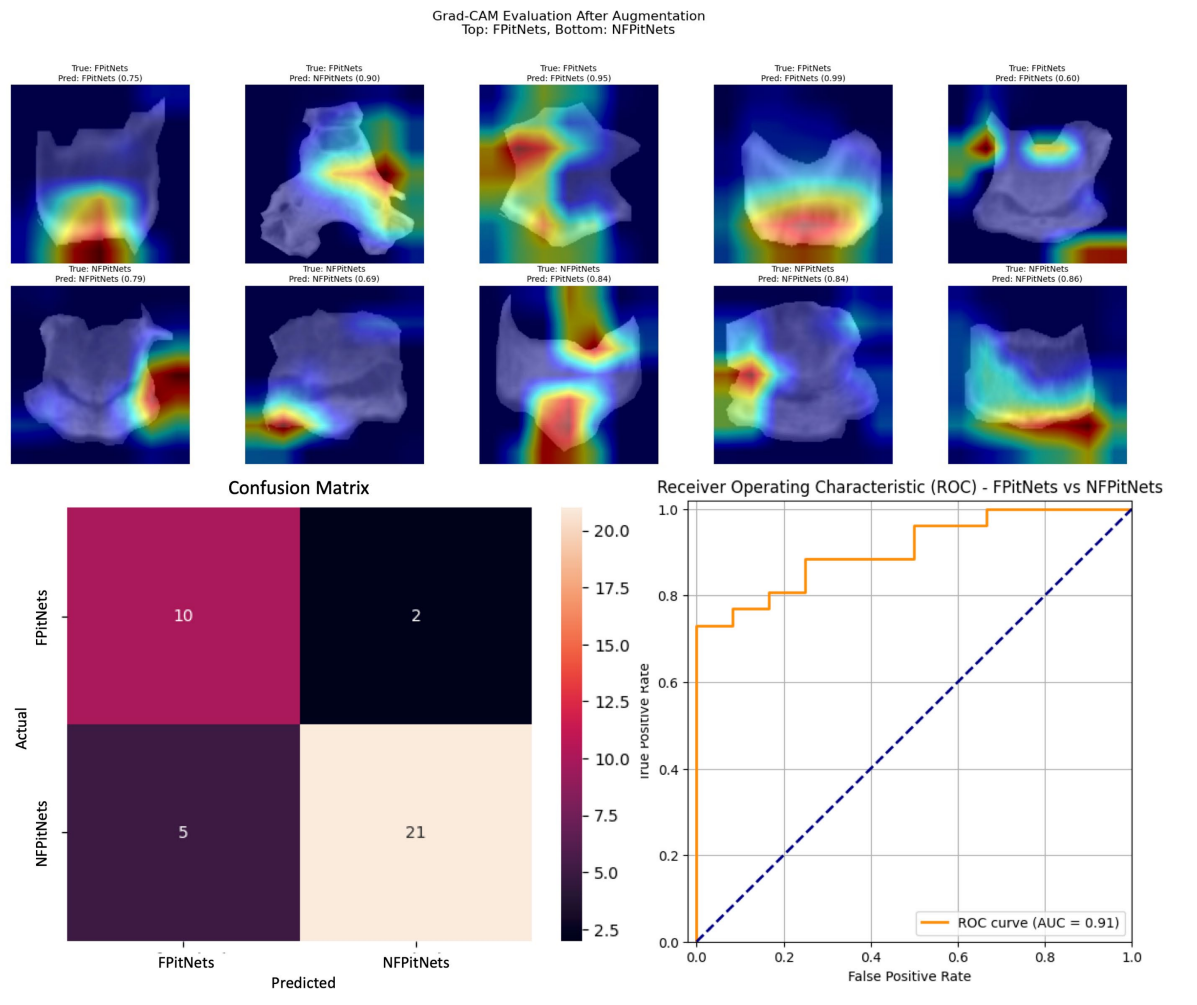
GRAD-CAM visualizations of CNN model emphasis in classification of groups, confusion matric, and ROC curve for Model 5 (coronal functional PitNET vs. nonfunctional PitNET).

**Figure 6:**
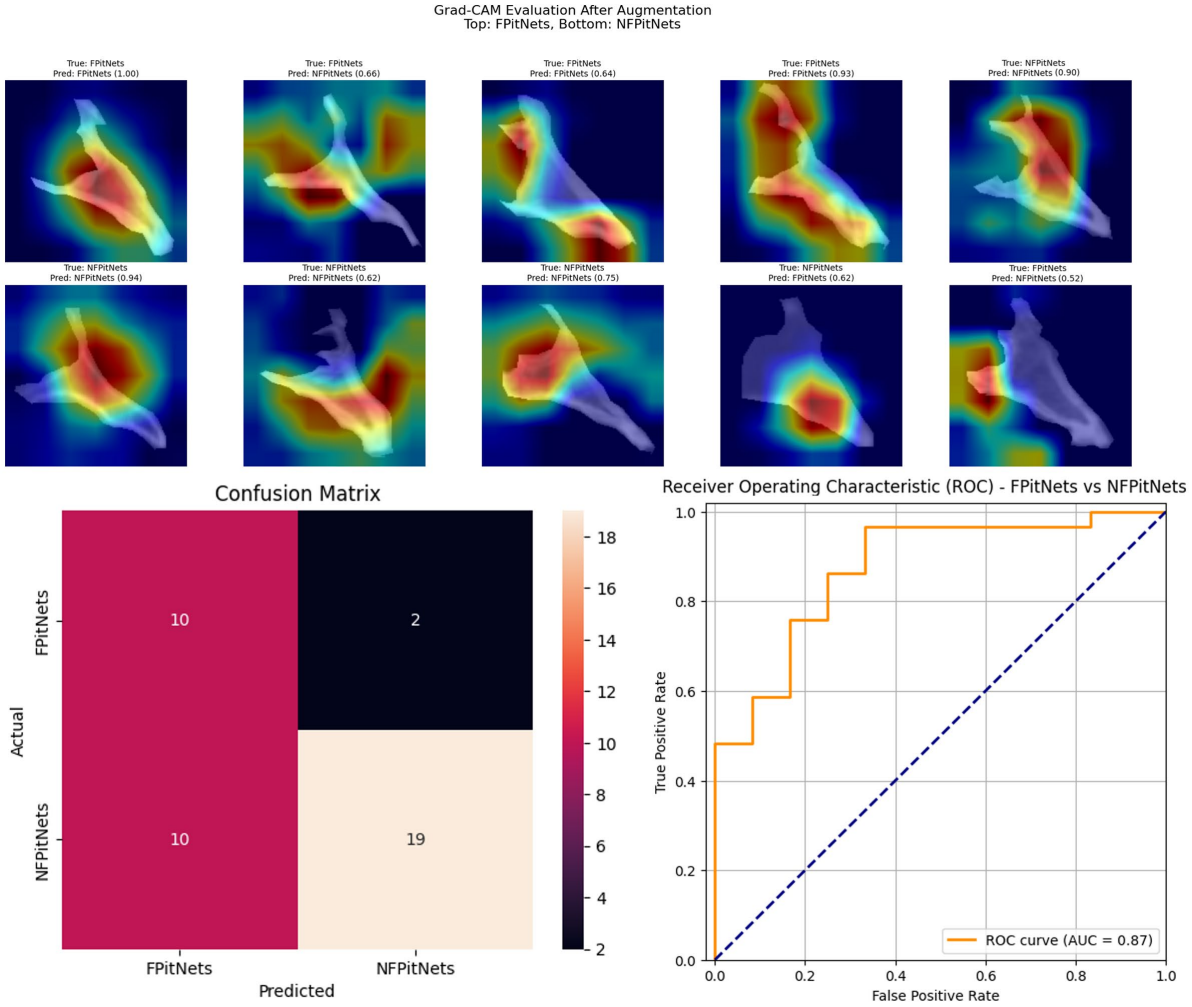
GRAD-CAM visualizations of CNN model emphasis in classification of groups, confusion matric, and ROC curve for Model 6 (sagittal functional PitNET vs. nonfunctional PitNET).

Models 4–6 evaluated presence of functionality/hormone secretion in presence of PitNet after training on images of the clivus across axial, coronal, or sagittal axes. Model 4 (axial non-functioning PitNET vs. functioning PitNET, Figure 4) had an accuracy of 0.95, AUC 0.97, and *F*_1_-scores of 0.96 (non-functioning PitNET) and 0.91 (functioning PitNET). Model 5 (coronal non-functioning PitNET vs. functioning PitNET, Figure 5) demonstrated accuracy 0.82, AUC 0.91, and *F*_1_-scores of 0.86 (non-functioning PitNET) and 0.74 (functioning PitNET). Model 6 (sagittal non-functioning PitNET vs. functioning PitNET, Figure 6) demonstrated accuracy of 0.71, AUC 0.87, and *F*_1_-scores of 0.76 (non-functioning PitNET) and 0.62 (functioning PitNET).

**Figure 7:**
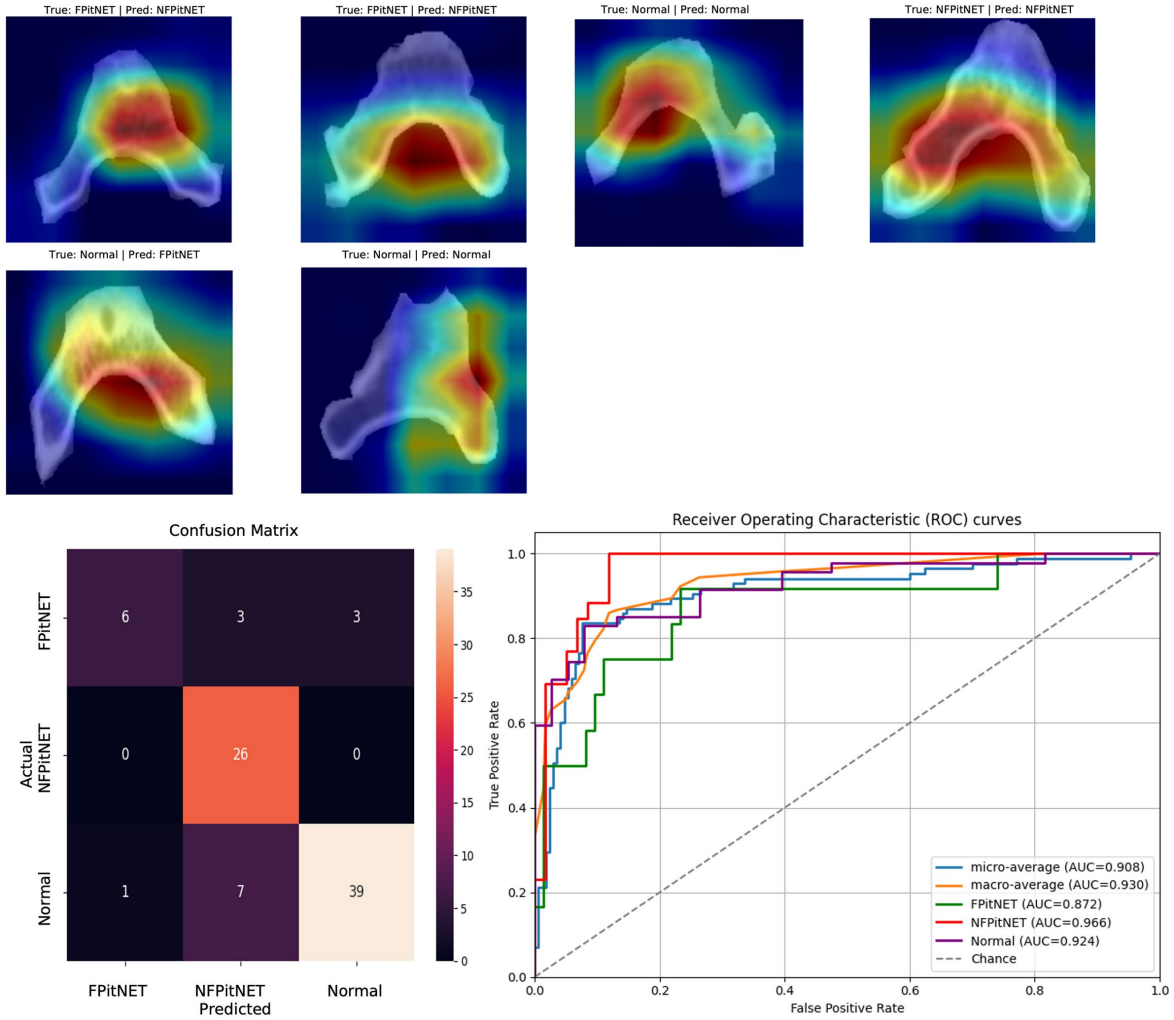
GRAD-CAM visualizations of CNN model emphasis in classification of groups, confusion matric, and ROC curve for Model 7 (axial functional PitNET vs. nonfunctional PitNET vs. normal).

**Figure 8:**
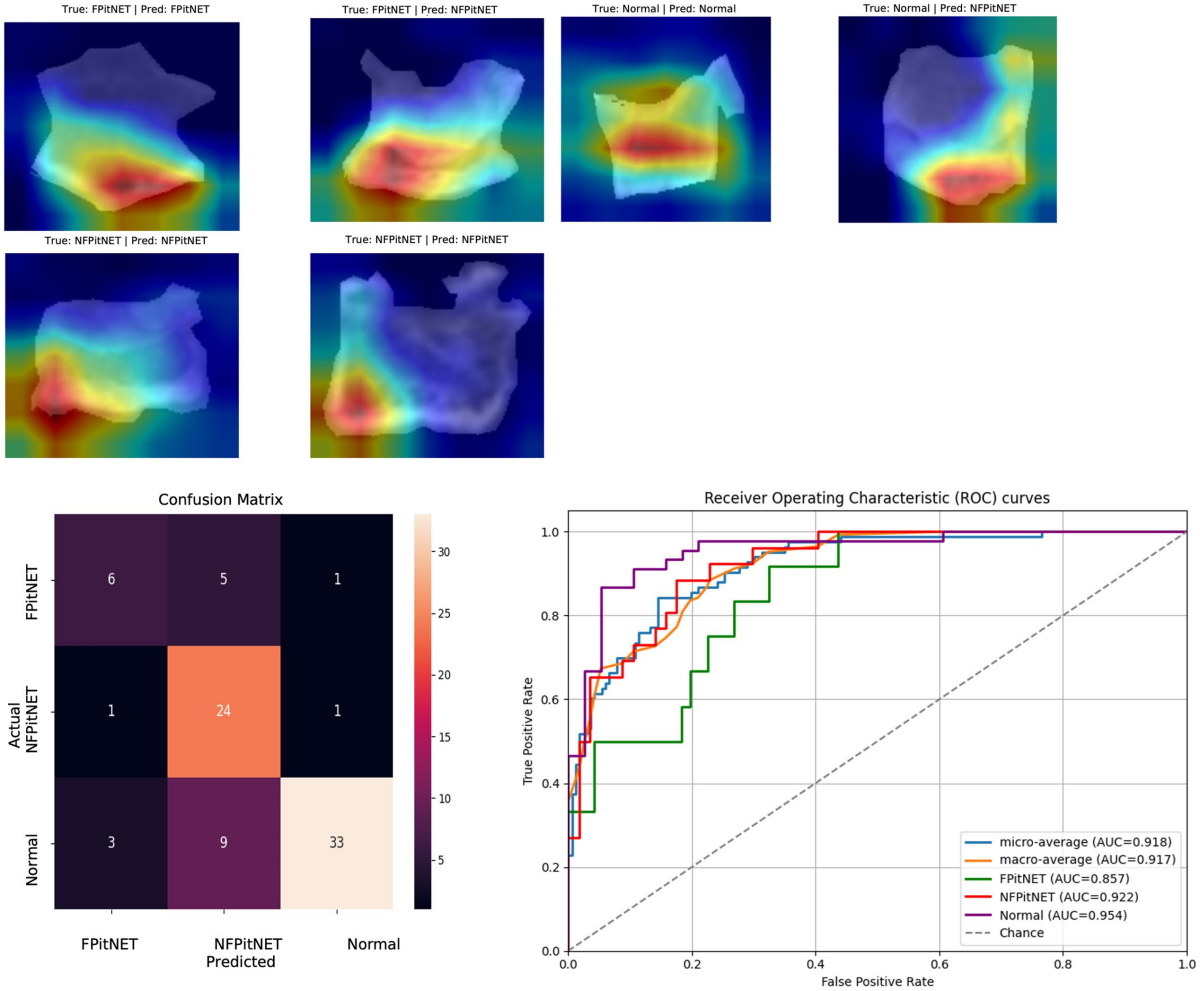
GRAD-CAM visualizations of CNN model emphasis in classification of groups, confusion matric, and ROC curve for Model 8 (coronal functional PitNET vs. nonfunctional PitNET vs. normal).

**Figure 9:**
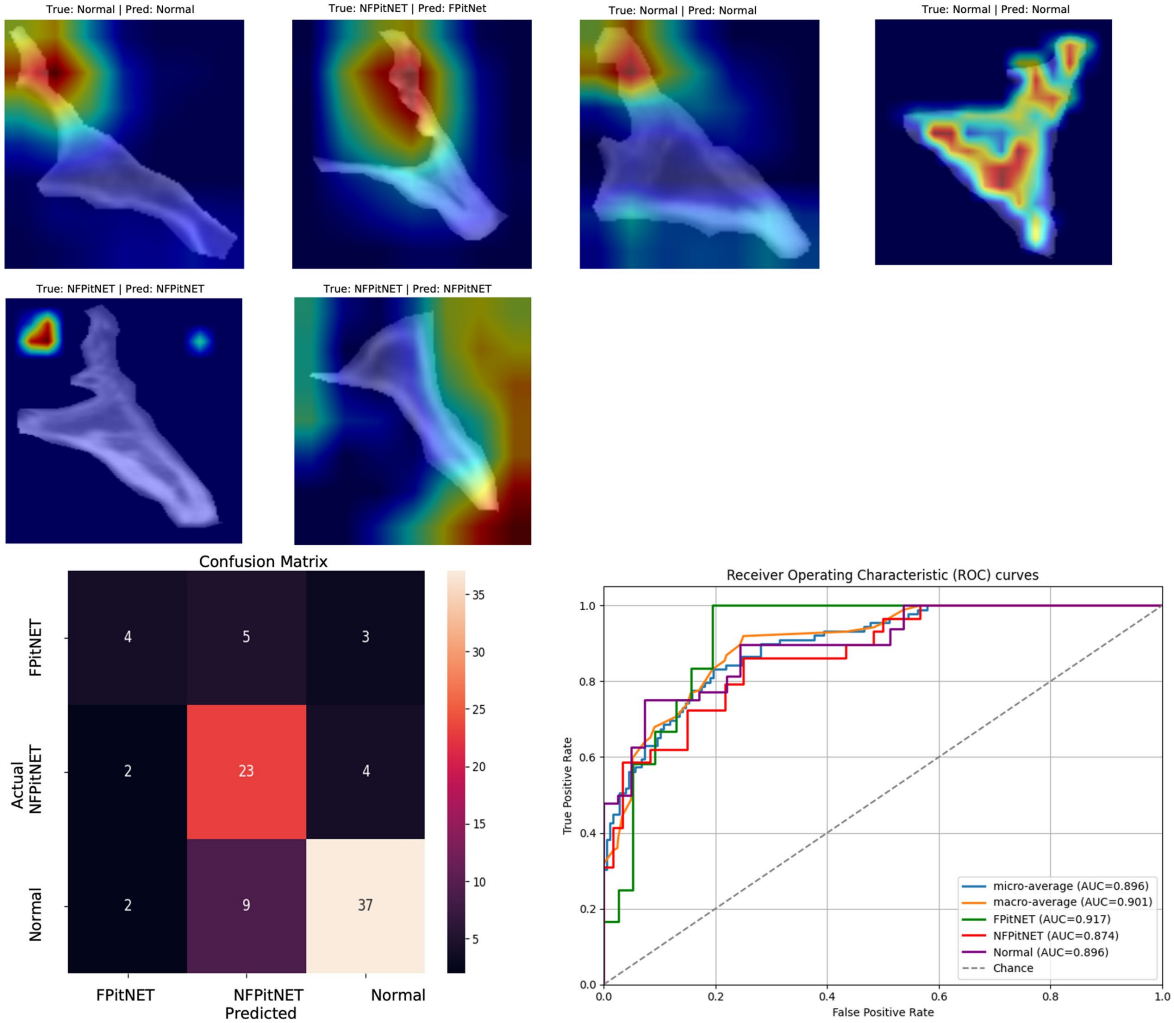
GRAD-CAM visualizations of CNN model emphasis in classification of groups, confusion matric, and ROC curve for Model 9 (sagittal functional PitNET vs. nonfunctional PitNET vs. normal).

Models 7–9 evaluated all groups (non-functioning PitNET vs. functioning PitNET vs. normal) across axial, coronal, or sagittal axes and evaluated for correct classification into each group. Model 7 (axial non-functioning PitNET vs. functioning PitNET vs. normal, Figure 7) had an accuracy of 0.83, AUC 0.92, *F*_1_-scores of 0.84 (non-functioning PitNET), 0.62 (functioning PitNET), and 0.87 (normal). Model 8 (coronal non-functioning PitNET vs. functioning PitNET vs. normal, Figure 8) demonstrated an accuracy of 0.76, AUC 0.91, *F*_1_-scores of 0.75 (non-functioning PitNET), 0.54 (functioning PitNET), and 0.82 (normal). Model 9 (sagittal non-functioning PitNET vs. functioning PitNET vs. normal, Figure 9) yielded accuracy of 0.72, AUC 0.89, *F*_1_-scores of 0.69 (non-functioning PitNET), 0.39 (functioning PitNET), and 0.80 (normal).

During risk of confounders analysis, there was a significant difference between the age and biological sex of the hormone secreting pituitary adenoma and non-functioning pituitary adenoma patients; the hormone secreting pituitary adenoma patients (mean age 43.7, SD = 18) were significantly (15.9 years, 95%-CI: 8.3–24, *p* < 0.001) younger than the non-functioning pituitary adenoma patients (average age 59.6 SD = 18), and the hormone secreting pituitary adenoma patients were significantly more likely to be female than the non-functioning pituitary adenoma patients (OR 3.3, 95%-CI: 1.3–8.3).

## Discussion

Pituitary neoplasms, including PitNETs, frequently expand the sella turcica and may invade into adjacent osseous structures including the sphenoid sinus inferiorly and/or the clivus posteriorly (1–5). Identification of sellar remodeling has previously assisted in diagnosing these tumors prior to more advanced imaging techniques such as computed tomography and magnetic resonance imaging (1). To the best of our knowledge, our report is the first to evaluate the potential role of AI/ML models to identify clival osseous changes in patients with and without PitNETs.

A series of convolutional neural networks (CNN) was created for this report. CNNs are deep learning algorithms composed of many layers transforming inputs/features (ex. images) to outputs (ex. absence/presence of tumor) (9). This report centered around use of two advanced deep-learning models (ResNet18 and Vision Transformer) to process inputs (bone window CT images of the clivus in non-functioning PitNET, functioning PitNET, and normal patients) and provide predictions on presence or absence of tumor and discernment of PitNET hormone secretion (7, 8). Additionally, to improve interpretability, gradient-weighted class activation mapping (GRAD-CAM) was used to generate heatmaps to demonstrate image regions influencing the models’ predictions (10).

In our report, accuracy of models varied by axis of image capture and outcome being evaluated (Table 2). Model performance was found to be consistently favorable for axial sequences Model 1 (axial PitNET vs. normal, accuracy 81%) and Model 4 (axial non-functioning PitNET vs. functioning PitNET, accuracy 95%), and Model 7 (axial non-functioning PitNET vs. functioning PitNET vs. normal, accuracy 83%). We attribute this performance difference to ability for the model to evaluate bilateral image features whereas sagittal sequences were taken at midline and only assess feature differences in the superior–inferior axis. Although this bilaterality of information is also available in coronal views, models consistently performed poorly compared to sagittal and axial sequences. Additionally, the model did fare favorably along the sagittal sequence in Model 3 (sagittal PitNET vs. normal, accuracy 81%), however, performance was less reliable for Model 6 (sagittal non-functioning PitNET vs. functioning PitNET, accuracy 71%) and Model 9 (sagittal non-functioning PitNET vs. functioning PitNET vs. normal, accuracy 72%); moreover, GRAD-CAM heat maps appeared less sporadic for Model 3 than Models 6 and 9 (Figures 3, 6, 9). Overall, our findings support the model’s ability to discern features along the anterior to posterior axis allow for more accurate prediction when compared to horizontal axis (Table 2). The coronal view model’s emphasis was on evaluating asymmetry from along the horizontal axis. The sagittal view model’s emphasis varied based on pathology (upper third in normal patients and middle third in tumor patients), and this performance was found to be inferior to their axial counterparts.

Li et al. (11) previously reported diagnostic accuracy of 94% and AUC 98% when evaluating for the presence or absence of pituitary microadenoma with a deep-learning based system trained on 1,520 magnetic resonance imaging scans. Another study by Qian et al. (12) also evaluated CNN performance with detecting pituitary adenomas based on MRI images and produced an overall accuracy of 91%. Although one of our models achieved similar accuracy and AUC (Model 4 axial non-functioning PitNET vs. functioning PitNET, accuracy 95%, AUC 97%), our models were trained on CT scans which are inherently lower resolution images. Moreover, our study only utilized bony windows of CT scans to evaluate if a deep learning model is capable of being trained on this limited information to discern the presence or absence of PitNETs and whether a PitNET may be hormone-secreting/functioning or not. Other models including Model 1 and Model 7 achieved acceptable accuracy and AUC percentages (axial PitNET vs. normal, accuracy 81%, AUC 87%; axial non-functioning PitNET vs. functioning PitNET vs. normal, accuracy 83%, AUC 92%) when considering this limited resolution and an overall dataset of 34 functioning PitNETs, 80 nonfunctioning PitNETs, and 129 non-tumor controls.

It is important to note that identifying an age and sex matched cohort to serve as anatomic control is challenging in a single centered small cohort of functioning pituitary adenoma patients because younger individuals are considerably less likely to undergo high-resolution CT imaging of the head compared to older patients. There are two likely reasons for this: (1) to avoid unnecessary radiation exposure in younger and female patients when the suspicion for anatomic brain abnormality is low, and (2) if the suspicion for anatomic brain abnormality high and further discovered on conventional CT, the patient can no longer serve as a control. Although, there was a significant gap between the age and sex of non-functioning pituitary adenoma patients and hormone secreting pituitary adenoma patients in this study, the published evidence in the literature is congruent with our patient population in that most hormone secreting pituitary adenoma patients are younger and more commonly female. Furthermore, the main goal of the machine learning model was to capture physiologic changes in the clivus that likely arise from a combination of patient demographics, including young age and female sex, rather than to adjust for them explicitly.

To the best of our knowledge, our CNN models are the first reported to assess for the presence or absence of PitNETs solely based on bony windows of the clivus bone. Although this proof-of-concept study yielded favorable results despite being limited to one image per patient per view, these tools are not currently ready for clinical application. However, future models may serve as diagnostic tools of substantial worth if implemented in developing countries with limited access to MR imaging as this model relies on single CT images, a modality more widely available than MRI (13).

### Limitations

Inherent limitations include a limited sample size of 134 PitNET patients, 129 non-tumor controls, and use of still bone-window CT images. Inherent limitations to deep-learning model training include overfitting, particularly in the setting of a smaller sample size. Although the models inherently execute separation of a dataset for evaluation purposes, additional external validation through multi-institutional collaboration may assist with assessing generalizable performance of these models. The typical functioning pituitary adenoma population is younger and more often female, which may introduce bias given known age- and sex-related morphologic differences. Model may not generalize to atypical functioning adenoma populations.

## Conclusion

Pituitary neoplasms frequently expand the sella turcica and may invade surrounding osseous structures including the sphenoid sinus and/or the clivus. To date, no reports have detailed use of a CNN to identify subtle osseous changes and potentially detect PitNETs based on CT bone windows alone. Several CNN models were created with average accuracies up to 81% in correct identification of PitNET versus control and 95% correct identification of functioning versus nonfunctioning PitNET. Although inherent limitations related to CNNs exist, these models provide evidence that CNNs may be trained to provide acceptable levels of accuracy with CT imaging, a modality more readily available than MRI. CNNs garner the potential to assist clinicians to detect PitNETs based on CT alone.

## Data Availability

The original contributions presented in the study are included in the article/supplementary material, further inquiries can be directed to the corresponding author.

## References

[1] Raghu ALB Flower HD Statham PFX Brennan PM Hughes MA. Sellar remodeling after surgery for nonfunctioning pituitary adenoma: intercarotid distance as a predictor of recurrence. J Neurol Surg B Skull Base. (2020) 81:579–84. doi: 10.1055/s-0039-1693700, 33134026 PMC7591359

[2] Campero A Martins C Yasuda A Rhoton AL Jr. Microsurgical anatomy of the diaphragma sellae and its role in directing the pattern of growth of pituitary adenomas. Neurosurgery. (2008) 62:717–23. doi: 10.1227/01.neu.0000317321.79106.37 18425018

[3] Tatreau JR Patel MR Shah RN McKinney KA Wheless SA Senior BA . Anatomical considerations for endoscopic endonasal skull base surgery in pediatric patients. Laryngoscope. (2010) 120:1730–7. doi: 10.1002/lary.20964, 20717950

[4] Velayudhan V Luttrull MD Naidich TP. "Sella turcica and pituitary gland". In: Imaging of the Brain. Philadelphia, PA: Elsevier (2012). p. 272–93.

[5] Wang J Wang R Lu Y Yao Y Qi S. Anatomical analysis on the lateral bone window of the sella turcica: a study on 530 adult dry skull base specimens. Int J Med Sci. (2014) 11:134–41. doi: 10.7150/ijms.7137, 24465158 PMC3894397

[6] Chang H Zhao K Qiu J Ji XJ Chen WG Li BY . Prediction of intraoperative cerebrospinal fluid leaks in endoscopic endonasal transsphenoidal pituitary surgery based on a deep neural network model trained with MRI images: a pilot study. Front Neurosci. (2023) 17:1203698. doi: 10.3389/fnins.2023.1203698, 37575298 PMC10413098

[7] Dosovitskiy A Beyer L Kolesnikov A Weissenborn D Zhai X Unterthiner T . (2020). An image is worth 16 × 16 words: transformers for image recognition at scale. *arXiv*. Available online at: 10.48550/arXiv.2010.11929. [Epub ahead of preprint]

[8] He K Zhang X Ren S Sun J. (2016). Deep residual learning for image recognition, 2016 IEEE Conference on Computer Vision and Pattern Recognition (CVPR). 770–778.

[9] Litjens G Kooi T Bejnordi BE Setio AAA Ciompi F Ghafoorian M . A survey on deep learning in medical image analysis. Med Image Anal. (2017) 42:60–88. doi: 10.1016/j.media.2017.07.005, 28778026

[10] Selvaraju RR Cogswell M Das A Vedantam R Parikh D Batra D. (2017). Grad-CAM: visual explanations from deep networks via gradient-based localization. 2017 IEEE International Conference on Computer Vision (ICCV). 618–626.

[11] Li Q Zhu Y Chen M Guo R Hu Q Lu Y . Development and validation of a deep learning algorithm to automatic detection of pituitary microadenoma from MRI. Front Med. (2021) 8:758690. doi: 10.3389/fmed.2021.758690, 34912820 PMC8666533

[12] Qian Y Qiu Y Li CC Wang ZY Cao BW Huang HX . A novel diagnostic method for pituitary adenoma based on magnetic resonance imaging using a convolutional neural network. Pituitary. (2020) 23:246–52. doi: 10.1007/s11102-020-01032-4, 32062801

[13] Khaing M Saw YM Than TM Mon AM Cho SM Saw TN . Geographic distribution and utilisation of CT and MRI services at public hospitals in Myanmar. BMC Health Serv Res. (2020) 20:742. doi: 10.1186/s12913-020-05610-x, 32787832 PMC7424658

